# Extending the use of *Centella asiatica* extract in poultry applications using distinct freeze-drying wall materials

**DOI:** 10.5713/ab.260300

**Published:** 2026-07-27

**Authors:** Khalil Ahmad, Worawan Panpipat, Manat Chaijan, Umaporn Uawisetwathana, Atikorn Panya, Natthaporn Phonsatta, Mingyu Yin, Roberto Castro-Muñoz, Chantira Wongnen

**Affiliations:** 1 School of Agricultural Technology and Food Industry, Walailak University, Nakhon Si Thammarat, Thailand; 2 Food Technology and Innovation Research Center of Excellence, School of Agricultural Technology and Food Industry, Walailak University, Nakhon Si Thammarat, Thailand; 3 Microarray Research Team, National Center for Genetic Engineering and Biotechnology (BIOTEC), National Science and Technology Development Agency (NSTDA), Pathum Thani, Thailand; 4 International Joint Research Center on Food Security (IJC-FOODSEC), Pathum Thani, Thailand; 5 Thailand Metabolomics Society, Bangkok, Thailand; 6 Food Biotechnology Research Unit, National Center for Genetic Engineering and Biotechnology (BIOTEC), Pathum Thani, Thailand; 7 College of Food Science and Technology, Shanghai Ocean University, Shanghai, China; 8 Shanghai Engineering Research Center of Aquatic-Product Processing and Preservation, Shanghai, China; 9 Gdansk University of Technology, Faculty of Civil and Environmental Engineering, Department of Sanitary Engineering, Gdansk, Poland; 10 Institute of Sustainable Processes, University of Valladolid, Valladolid, Spain

**Keywords:** Antioxidant Activity, *Centella asiatica*, Gastrointestinal Release, Microencapsulation, Phytogenic Feed Additive, Protein-based Carriers

## Abstract

**Objective:**

*Centella asiatica* is a promising phytogenic feed additive for poultry because of its bioactive compounds; however, its heat instability and poor palatability limit direct feed incorporation. Although microencapsulation can enhance stability and controlled release, there have been few comparative studies of wall materials for poultry applications.

**Methods:**

The leaf powder ethanolic extract of *Centella asiatica* was freeze-dried and microencapsulated with maltodextrin+gum arabic (MD+GA), polyethylene glycol, albumen, gelatin, and sodium alginate across four treatments (T1–T4, core-to-wall ratios 1:10 to 1:4). Encapsulation efficiency (EE %), total phenolic content (TPC), DPPH antioxidant activity, intestinal digestibility, thermal stability (Tm), water activity (aw), and morphology (scanning electron microscopy [SEM] and Fourier-transform infrared spectroscopy) were evaluated. The data was analyzed using analysis of variance and Tukey’s honest significant honest significant difference (p<0.05).

**Results:**

MD+GA exhibited the greatest EE % (98.67±0.47%, T1), followed by albumen (94.14±0.94%, T2) and gelatin (85.12±0.85%, T3). Gelatin and albumen showed the greastest TPC (35.81 and 36.44 mg GAE/g) and DPPH activity (27.51±0.41%, T4; 22.07±0.33%, T4), whereas MD+GA showed moderate values (32.73 mg/g; 20.50±0.31%, T4). MD+GA and gelatin had good intestinal digestibility (82.38±1.24%, T1 and 93.21±1.40%, T3, respectively). Albumen (a_w 0.34±0.01, T2) and gelatin (0.39±0.01, T3) were effective moisture controllers, whereas MD+GA was moderate (0.67±0.01, T3). SEM revealed smooth, spherical protein-based microcapsules and rougher polysaccharide-based microcapsules.

**Conclusion:**

MD+GA provided optimal EE % and intestinal release compared with protein-based materials, while also maintaining good phenolic retention and antioxidant activity, indicating their suitability for antibiotic-free poultry feed formulations.

## INTRODUCTION

Globally, the poultry industry contributes to food security by providing affordable, efficient access to high-quality animal protein. It contributes to up to 40% of meat production worldwide and has become essential in the diet of human populations [1]. Between 1960 and 2000, the share of total meat production doubled from 15% to 30%, and by 2020, global poultry production had reached 137 million tons, demonstrating its high production growth rate [2]. Currently, one-third of the world’s population consumes animal-sourced poultry protein. The prospects of global food systems also demonstrate that by 2030, poultry will contribute 41% of the world’s total meat consumption, which solidifies its position as an inevitable global food system entity [3]. The increasing global demand for and consumer preference for antibiotic-free, nutritious poultry meat have spurred interest in production practices that enhance poultry health and productivity [4–6]. Phytogenic feed additives (PFAs), derived from plant-based compounds, are increasingly used as alternatives to antibiotics in poultry production because of their antimicrobial, antioxidant, and immune-boosting properties, which address consumer demand for antibiotic-free and nutritious meat [7,8].

*Centella asiatica* (L.) is a traditional medicinal herb from Southeast Asia that contains major bioactive compounds, such as triterpenoids, flavonoids, and phenolic acids [9]. The chemical compounds in this plant exhibit anti-inflammatory, antioxidant, neuroprotective, and antimicrobial activities that may improve broiler health during periods of stress [10]. Asiaticoside and asiatic acid specifically exhibit the potential to regulate immune and inflammatory pathways, in terms of their relevance to poultry health [11]. Recent findings have demonstrated that asiatic acid inhibits nuclear factor kappa B (NF-κB), which subsequently lowers the expression of pro-inflammatory cytokines such as TNF-α and IL-6, and enhances the expression of antioxidant enzymes such as superoxide dismutase (SOD) and catalase. These mechanisms play an important role in reducing enteric stress and systemic inflammation, which are typical features of intensive broiler chicken production [12].

Despite these prominent properties, the direct addition of *C. asiatica* extract to poultry feed is limited because of several challenges and functional barriers, including heat instability during production, sensitivity to gastric pH, and an unpleasant taste, which could reduce its effects and lower acceptance [13]. These limitations may decrease the efficacy of bioactive compounds when administered as part of the feed. Microencapsulation is a prominent method for enhancing the performance of bioactive compounds in poultry feed by improving their stability, bioavailability, and controlled release, thereby addressing issues such as degradation and volatility [14,15]. Encapsulation is a technique that entraps one material within another, resulting in particles that vary in size from a few nanometers to a few millimeters. The enclosed material is known as an active agent, payload phase, or internal phase. The material surrounding the encapsulant is referred to as the matrix or exterior phase [16]. Using these approaches, sensitive substances are protected against degradation, shelf life is improved (Figure 1), unwanted flavors can be hidden, and the supplement can be released at the appropriate time in the gut [17]. Many feed producers prefer freeze-drying to find a solution for encapsulation because it is compatible with sensitive compounds, cost-effective, and can be used on a large scale when the parameters are adjusted for the best outcome.

The encapsulation results were highly dependent on the selection of coating and shell materials. For their biocompatibility, ability to act as emulsifiers, and protective properties, maltodextrin (MD), gum arabic (GA), and sodium alginate (S. Alginate) are oft en used as polymer additives in drug development [18]. They affect the physical structure of the nanoparticles, the amount of product captured in each particle, and the speed of their release. Although the encapsulation of plant extracts with nutraceutical and pharmaceutical applications is well known, the process of encapsulation of *C. asiatica* specifically for use in poultry nutrition has been sparse, and comparative analysis evaluating the differences in the characteristics of encapsulation and gastrointestinal release profile of its bioactive compounds with the use of different food-grade wall materials has not yet been conducted thus far [19]. Furthermore, release studies in simulated poultry gastrointestinal environments (pH 3–6, enzymatic digestion) have rarely been conducted, and there is a gap in the knowledge regarding *in vivo* functionality. Therefore, the present study aims to fill this gap by designing freeze–dried microcapsules of the *C. asiatica* ethanolic extract using an assortment of wall materials and determining their encapsulation efficacy, antioxidant potential, thermostability (differential scanning calorimetry [DSC]), and gastrointestinal discharge profile. The results should contribute to the logical formulation of phytogenic additives for feedstuff that are thermally resistant, biologically active, consistent with the sustainability of modern poultry producers, and free of all antibiotics.

## MATERIALS AND METHODS

### Experimental site

The experiment was conducted at Walailak University, Nakhon Si Thammarat, Thailand (latitude: 8°38′16.59′ ‘N, longitude: 99°53′29.99′’ E).

### Plant extraction and preparation of microcapsules

Fresh *C. asiatica* plants were procured from a local farm in Tha Sala, Nakhon Si Thammarat, Thailand. The plant material was thoroughly rinsed with running tap water to remove surface debris. Drying was performed at 45°C and 2,500 W using a microwave oven until the moisture level was reduced to less than 10%. The dried material was ground using a Hammer Mill (2-mm sieve) and stored at 4°C for further processing.

Extraction was performed using 50% ethanol in a 1:20 (w/v) ratio [20]. The mixture was sonicated for 30 min at 40 kW and stirred at 800 rpm for 24 h using a sonicator (Sonics & Materials). After incubation, the coarse plant debris was filtered using muslin cloth, and fine filtration was performed using Whatman No. 1 filter paper. The resulting solution was concentrated using a rotary evaporator (BUCHI Rotavapor R-114). The concentrated solution was stored at 4°C and freeze-dried at −65°C for 48 h using a freeze dryer (Spring Green Evolution) with a vacuum pressure of 0.025 mbar to obtain a powdered extract that was used for encapsulation.

### Wall materials and encapsulation procedure

Five different types of wall materials from different classes were used for encapsulation. The ingredients included MD, GA (50:50 w/w), polyethylene glycol (PEG), egg albumen, gelatin, and S. Alginate. Four core-to-wall ratios were used for each encapsulation material: T1 = 1:10, T2 = 1:8, T3 = 1:6, and T4 = 1:4. as presented in the Supplement 1.

“T5 represented wall material only and was used as a blank/reference treatment for selected physicochemical characterization. Therefore, T5 was excluded from extract-dependent analyses, including DPPH radical-scavenging activity, encapsulation efficiency (EE), intestinal digestibility, and thermal transition temperature.”

Each wall and core material was dissolved in distilled water (1 mg/mL) and stirred until it was thoroughly dissolved to obtain a solution. The mixture was sonicated at 40 kW for 15 min to ensure its homogeneity. Sonication was followed by lyophilization in a Labconco Free Zone 12 plus at −80°C for 48 h. For S. Alginate encapsulation, the S. Alginate solution was added to the calcium solution to prepare the beads, which were then freeze-dried, as previously described.

### Characterization of microencapsulated *Centella asiatica* extract

#### Total phenolic content

The total phenolic content (TPC) was determined using the Folin-Ciocalteu method. The sample was taken in an amount of 100 μL and added to 500 μL of 10% Folin-Ciocalteu reagent and incubated for 5 min. This was followed by the addition of 400 μL of 7.5% Na_2_CO_3_, and the mixture was allowed to stand in the dark at room temperature for 30 min. A UV-Vis spectrophotometer (Shimadzu UV-1800) was used to determine absorbance at a wavelength of 765 nm. A gallic acid standard curve was plotted and TPC was determined as mg gallic acid equivalents (GAE) per gram dry weight [21].


(1) $$\text{TPC }(\text{mg QE}/\text{g})=\frac{\left(\text{C}\times \text{V}\right)}{M}$$

where C is the concentration obtained from the calibration curve (mg/mL), V is the volume of the extract (mL), and M is the mass of the sample (g).

### DPPH radical scavenging activity

The DPPH radical-scavenging method was used to determine the antioxidant activity. Briefly, 0.1 mL of the extract was added to 3.9 mL (0.1 mM) of DPPH methanolic solution and incubated in the dark at room temperature for 30 min. The absorbance was measured at 517 nm using a UV-Vis spectrophotometer.


(2) $$\%\;\text{Inhibition}=([{\text{A}}^{0}-{\text{A}}_{\text{s}}]/{\text{A}}_{0})\times 100$$

where A_0_ and A_s_ are the absorbance values of control and sample, respectively.

### *In vitro* release profile

*In vitro* release studies simulated the gastrointestinal conditions in poultry using two sequential digestion phases. For the gizzard phase, 100 mg of microcapsules was suspended in 50 mL of buffer pH 3.0 containing 1.5 mg/mL pepsin (P7000; Sigma-Aldrich) and incubated at 37°C for 45 min. The mixture was then centrifuged at 3,000×g for 10 min. The pellet was resuspended in 50 mL buffer (pH 7.0 buffer containing 2 mg/mL pancreatin (P7545; Sigma-Aldrich) and incubated for 2 h at 37°C (intestinal phase). The supernatants from each stage were collected and analyzed for phenolic release using the TPC method [22].

The percentage of phenolic compounds released was calculated as follows:


(3) %=(Released phenolics/Total phenolics)×100.

### Fourier-transform infrared spectroscopy

Fourier-transform infrared spectroscopy (FTIR) was performed using a Bruker Tensor 27 instrument with an ATR accessory. The spectra were detected within 4,000–400 cm^−1^, ranging from a resolution of 4 cm^−1^ to 32 scans per sample. The functional groups and molecular interactions between *C. asiatica* extract and the wall materials were identified using FTIR.

### Differential scanning calorimetry

DSC was performed on a PerkinElmer DSC 6000 instrument. A sample weighing approximately 5–10 mg was sealed in aluminum pans and heated between 20°C and 200°C at a 10°C/min rate and nitrogen flow rate of 20 mL/min. Thermal transition temperature (Tm) was recorded to understand the thermal behavior of the microcapsules.

### Scanning electron microscopy

Scanning electron microscopy (SEM; JEOL JSM-IT300) was used to study the surface morphology of the microcapsules. The samples were gold-coated and examined at magnifications of 100–2,500×. Sample interference in SEM analysis was used to provide information on the smoothness of the surfaces, wall integrity, and encapsulation uniformity.

### Statistical analysis

All results are expressed as the mean±standard deviation (SD). Data were analyzed using one-way analysis of variance (ANOVA). When significant differences were detected, treatment means were separated using Tukey’s honest significant difference (HSD) test at p<0.05. Statistical computations were performed in RStudio (ver. 4.2.1) using the agricolae package for ANOVA and post-hoc analysis and the car and dplyr packages for additional statistical support and data handling. Data visualization was conducted using Python (ver. 3.10.13) with libraries such as Matplotlib, Seaborn, and Pandas.

## RESULTS

### Encapsulation efficiency and total phenolic content/DPPH trends

The EE % and DPPH free radical scavenging activity varied significantly across the formulations, whereas the TPC varied substantially. The TPC, DPPH antioxidant activity, and EE % analyses highlighted the performance of different encapsulating agents (MD+GA, PEG, albumen, gelatin, and S. Alginate) across treatments, with statistical significance determined by ANOVA and Tukey’s HSD tests (p<0.05). For TPC, gelatin exhibited the highest mean phenolic content (35.81 mg GAE/g), followed by albumen (36.44 mg GAE/g), MD+GA (32.73 mg GAE/g), PEG (26.17 mg GAE/g), and S. Alginate (17.27 mg GAE/g), indicating superior phenolic retention in protein-based materials. The effects of wall material (F = 912.09, p<2e-16) and treatment level (F = 286.58, p<2e-16) on TPC were highly significant, with a good interaction (F = 11.85, p = 1.12e-09), suggesting that phenolic retention varies with specific material-treatment combinations, notably with gelatin-T4 (Figure 2). The DPPH results in Table 1 further detail treatment-specific performance: in T4, gelatin (27.51±0.41%, a), albumen (22.07±0.33%, a), MD+GA (20.50±0.31%, a), PEG (21.74±0.33%, a), and extract (21.50±0.32%, a) showed peak antioxidant activity, while S. Alginate remained the lowest (9.10±0.14%, a). In T1–T3, gelatin and albumen consistently outperformed the others, with T3 values of 25.79±0.39% (b) and 20.58±0.31% (b), respectively. For EE % (Supplement 2 and Figure 3), MD+GA achieved the highest mean efficiency (97.89%), followed by albumen (86.55%) and gelatin (83.77%), indicating robust encapsulation capabilities, whereas PEG (30.55%) and S. Alginate (71.23%) showed moderate to low efficiencies, likely owing to weaker molecular interactions with phenolic compounds. Supplement 2 shows that the peak of MD+GA was at T1 (98.67±0.47%, a), albumin at T2 (94.14±0.94%, a), and gelatin in T3/T4 (85.12±0.85%, a; 84.16±0.84%, a), with the wall material effect being highly significant (F = 53.677, p = 1.47e-07), unlike the non-significant treatment effect (F = 2.047, p = 0.161). These findings underscore the superior EE of MD+GA, driven by wall material properties and the effectiveness of gelatin and albumen in retaining phenolic content and antioxidant activity, particularly in T4, making them ideal for applications requiring sustained bioactive release.

### Correlation insights among key parameters

Correlation analysis demonstrated a statistically significant positive relationship (r = 0.819, p<0.001) between TPC and DPPH activity, indicating that an increase in phenolic content directly implies an increase in the antioxidant capacity (Supplement 3). TPC was moderately positively correlated with water activity (a_w_) (r = 0.597) and DPPH and aw (r = 0.483), indicating that moisture conditions can support the stability and release of active compounds. Remarkably, there was a moderate and statistically significant negative relationship between TPC and gizzard digestibility (r = −0.643), which might be impeded, presumably by extraordinarily rigid matrices or complexes between phenolics and proteins in some digestive environments in which phenolic compounds are released. Furthermore, a_w_ and gizzard digestibility exhibited the strongest negative relationship (r = −0.810), with water mobility being a decisive factor in altering digestibility levels. Most variables exhibited low or moderate correlations with EE %, indicating that, although EE % is reliable for determining compound retention, it may not strongly influence the release or bioactivity data.

### Intestine digestibility

Digestibility of microcapsules in a simulated intestinal phase across four treatments (T1–T4) with encapsulating agents MD+GA, PEG, albumen, gelatin, and alginate, with significant differences indicated by superscripts (ANOVA, Tukey’s HSD, p<0.05) (Table 2). In Treatment T1, MD+GA exhibited a high digestibility of 82.38±1.24% (a), which was comparable to albumen (82.75±1.24%, b) and surpassed that of gelatin (80.56±1.21%, b), PEG (71.91±1.08%, a), and S. Alginate (74.22±1.11%, b), highlighting its efficiency in nutrient release during intestinal digestion. The high digestibility of MD+GA in T1 likely stems from favorable conditions such as optimal pH, enzyme activity, or formulation properties, which enhance its interaction with intestinal enzymes and solubility. In T2, S. Alginate showed good digestibility (79.94±1.20%, a), followed by MD+GA (76.88±1.15%, b) and albumen (76.92± 1.15%, c). T3 recorded gelatin peaking at 93.21±1.40% (a) and albumen at 84.59±1.27% (a), indicating strong enzymatic hydrolysis for protein-based materials, whereas MD+GA (72.46±1.09%, c) remained moderate. In T4, all agents showed lower digestibility, with MD+GA at 64.97±0.97% (d), PEG at 63.85±0.96% (c), and albumen at 71.14±1.07% (d). The superior performance of MD+GA in T1, comparable to that of protein-based albumen and gelatin, underscores the compatibility of its chemical structure with intestinal digestion, making it a promising coating material for controlled nutrient release.

### Thermal stability (differential scanning calorimetry)

The results indicated significant variations in thermal stability among the treatments and encapsulating agents, as evidenced by the mean±SD values and superscripts denoting statistical differences (ANOVA and Tukey’s HSD test, p<0.05). Table 2 shows the thermal transition temperature (T_m_, °C) of the microcapsules across treatments with the encapsulating agents MD+GA, PEG, albumen, gelatin, and alginate. In T1, MD+ GA had a T_m_ of 74.48±1.12°C, which was higher than that of albumen (71.48±1.07°C) but lower than that of gelatin (80.17±1.20°C), PEG (92.76±1.39°C), and S. Alginate (104.55±1.57°C), indicating good thermal stability and processability for controlled-release applications. S. Alginate (T1, T_m_ = 104.55±1.57°C, ΔH = 6.42 mJ) and PEG (T1, T_m_ = 92.76±1.39°C, ΔH = 4.59 mJ) exhibit superior thermal stability. In T2, the highest T_m_ was observed for gelatin (93.22± 1.40°C), whereas in T4, MD+GA peaked at 103.44±1.55°C. Albumen and gelatin showed moderate T_m_ values in T3 (73.78±1.11°C and 81.62±1.22°C, respectively). Statistical analysis (ANOVA, Tukey’s HSD, p<0.05) confirmed significant differences, with superscripts (a, b, c, and d) highlighting distinct thermal profiles, guiding the selection of the guiding material for microencapsulation (Table 2).

### Functional group validation via Fourier-transform infrared spectroscopy

*C. asiatica* FTIR microcapsules with different wall materials (Figure 4) exhibited clear spectral variations and peak shifts, reflecting differences in intermolecular interactions. In the MD+GA group, all samples showed a broad O–H stretching band, with the strongest intensity in T5, indicating enhanced hydrogen bonding between polysaccharide hydroxyl groups and phenolic compounds. T4 and T5 exhibited pronounced peaks at ~1,000–1,100 cm^−1^ (C–O–C stretching), suggesting strengthened glycosidic linkages and improved matrix stabilization. In the PEG group, deeper O–H bands were observed in T2 and T3, with notable shifts in the fingerprint region (900–1,200 cm^−1^), particularly in T1 and T5, indicating polymer–bioactive interactions and possible chain rearrangement within the matrix. Albumen-based formulations showed high variability, especially T5, with broader and deeper absorption near ~3,300 cm^−1^ and distorted transmittance between 1,500–1,000 cm^−1^, suggesting strong protein–polyphenol interactions mediated by hydrogen bonding and electrostatic forces. The gelatin group exhibited similar profiles, with increased intensity in T4 and T5 within the amide I and II regions (~1,650 and 1,540 cm^−1^), reflecting enhanced amide bonding interactions and structural reorganization of the protein network. S. Alginate spectra demonstrated a distinct pattern with sharp peaks at 1,030–1,100 cm^−1^ (T1–T4) and the lowest transmittance in the 3,200–3,400 cm^−1^ region for T5, indicating stronger hydroxyl interactions and ionic crosslinking behavior typical of alginate systems. These spectral differences confirm that the encapsulation systems involve varying degrees of hydrogen bonding, electrostatic interactions, and matrix rearrangement, influencing the stability and interaction of *C. asiatica* bioactives.

### Morphological analysis of the generated microcapsules via scanning electron microscopy

Morphological analysis of the encapsulated formulations using SEM revealed different structural features, depending on the composition of the wall material. Smooth, spherical microcapsules with uniform surfaces were observed on protein-based wall materials, such as albumen and gelatin, suggesting well-developed encapsulation structures and optimum wall intactness (Figure 5). In contrast, polysaccharide-based materials demonstrate structural compromise to varying extents. By contrast, polysaccharide-based materials exhibit varying degrees of structural compromise. MD-GA (MGA) formed partially distorted spheres with intermediate surface roughness, exhibited moderate EE, and S. Alginate formed collapsed or shrunken spheres with porous, and cracked surfaces, indicating poor structural stability (Figure 5). These results highlight the film-forming and structural advantages of protein-based materials over polysaccharide or synthetic polymer alternatives, all of which have morphological defects that would undermine EE, core protection, and controlled release performance. These findings imply that the use of protein-based wall materials is essential in situations where precise morphology and continued release are essential, and that the formulation based on polysaccharides and polymers can be altered to increase its structural stability. This is because the combination of protein and polysaccharide results in heightened electrostatic interactions between the two components, resulting in a more stable particle size and a regular morphology. Moreover, the combination of a polysaccharide with the protein enhanced EE and prolonged the storage period of the encapsulated components [23].

Morphological analysis of the encapsulated formulations using SEM revealed distinct structural features depending on the composition of the wall material. Overall, the particles exhibited a porous, sheet-like and amorphous morphology characteristic of freeze-dried systems, rather than discrete spherical microcapsules. Protein-based wall materials (albumen and gelatin) showed relatively more continuous and compact matrix structures with fewer cracks and better surface integrity, indicating improved encapsulation behavior (Figure 5). In contrast, polysaccharide-based materials displayed less uniform structures with higher porosity, structural collapse, and surface irregularities. The MD–GA system formed moderately cohesive matrices with some surface roughness, whereas S. Alginate exhibited more pronounced shrinkage, cracking, and structural deformation. These differences reflect variations in film-forming ability and matrix stability among wall materials. Overall, protein-based systems demonstrated comparatively better structural integrity, which may contribute to enhanced core protection and controlled release performance, while polysaccharide-based systems may require further optimization to improve stability.

### Water activity and color properties

Water activity (a_w_) and color properties (*L*, *a*, *b*) of *C. asiatica* microcapsules across treatments (T1–T5) for encapsulating agents MD+GA, PEG, albumen, gelatin, and alginate, with significant differences indicated by superscripts (ANOVA, Tukey’s HSD, p<0.05) (Tables 3, 4). The albumen-based microcapsules in T2 exhibited the lowest and least variable a_w_ (0.34±0.01, d), indicating excellent moisture control, paired with high lightness (L: 76.86–79.99) and a green-leaning color (a: −0.6, −0.46), suggesting visually stable and appealing microcapsules. Gelatin in T3 exhibited a similarly low a_w_ (0.39±0.01, b) and favorable color (L: 61.5–79.46), confirming its efficacy as a protein-based wall material with good moisture stability and aesthetics. MD+GA in T1 and T3 displayed moderate a_w_ (0.66±0.01, b; 0.67±0.01, a) with satisfactory color (L: 79.99, a: −0.6, b: 15.96), indicating adequate but less robust moisture barrier properties compared to protein-based materials. PEG in T4 had the highest a_w_ (0.88±0.01, b), reflecting poor moisture resistance and a darker reddish-yellow color (L: 60.45, a: 1.37, b: 24.18), suggesting undesirable color changes. S. Alginate in T4 shows the lowest a_w_ (0.10±0.00, d) and highest lightness (L: 86.62, b: 2.7), indicating superior dryness but potential structural weaknesses. These results highlight that protein-based albumen and gelatin offer optimal moisture protection and aesthetic stability, whereas MD+GA provides a moderate performance. PEG and S. Alginate showed trade-offs between moisture control and color stability, guiding their application in microcapsule formulations.

## DISCUSSION

The encapsulation of *C. asiatica* ethanolic extract with different wall materials, including MGA, PEG albumen, gelatin, and S. Alginate, resulted in significant differences in the (EE %, TPC, antioxidant activity (DPPH), *in vitro* release profiles, and morphological characteristics. These variations can be attributed to the physicochemical characteristics of the wall materials, which can determine the stability, bioavailability, and controlled release of the phytoconstituents, as supported by previous encapsulation research reports [24]. The morphology observed is strongly influenced by freeze-drying conditions and matrix interactions [25]. During freezing, ice crystals act as templates and their sublimation generates porous structures [26], where freezing rate and formulation properties (e.g., solids and viscosity) control pore size and structural collapse, leading to amorphous, sheet-like morphologies typical of freeze-dried systems rather than discrete microspheres. In parallel, differences among wall materials arise from their molecular interactions, where polysaccharide systems form more porous, glassy matrices due to weaker interactions [27], whereas protein-based systems form more compact and continuous structures through stronger intermolecular bonding, resulting in improved structural integrity [28].

MGA exhibited an exceptional EE of (97.39±1.09), surpassing values reported for similar polysaccharide-based systems (GA/MD for pepper oil), underscoring its superior ability to retain *C. asiatica* bioactive compounds and phenolic acids [29]. This high EE can be ascribed to the synergistic effect between MD, a low-molecular-weight carbohydrate that provides a protective non-crystalline glassy structure, and GA, a natural polysaccharide with emulsifying and film-forming capabilities [18,30]. The carbohydrate matrix forms a semi-permeable layer that reduces oxidative loss during the extraction step due to freeze-drying, and the GA proteinaceous component improves the interfacial stability, allowing GA to trap hydrophobic and polar phytoconstituents. The moderate TPC of 32.73 mg GAE/g and DPPH inhibition of 17.75% indicate a strong retention of antioxidants, although both of these results were inferior to those of the protein-based materials, presumably because of the lighter, more porous nature of the polysaccharide mixture. SEM indicated roughness-intermediateness with implications of greater bounding-up versus permeability and possible controlled-releasing morphology. This dual mechanism ensures a high retention rate, as evidenced by the EE surpassing protein-based materials such as albumen (86.55%) and gelatin (83.77%), despite the denser matrices of the latter. Considering the amphiphilicity, biocompatibility, and nutritional aspects of proteins, they remain valuable; however, the high EE of MGA aligns with its cost-effective scalability, which is a key advantage in poultry feed applications.

The release profile of MGA in the simulated gastrointestinal tract is beneficial for broiler production. This high intestinal digestibility indicates biphasic release kinetics of the first burst release of surface-bound phenolics, followed by sustained diffusion due to the high susceptibility of the polysaccharide matrix to enzymatic hydrolysis [31]. Amylases and cellulases released in the broiler gut target glycosidic linkages within MD and GA, as shown in the FTIR spectral bands at 1,000–1,100 cm^−1^ (C-O-C stretching), which facilitates the erosion of the matrix at intestinal pH, 6–7. This aligns with the observed 82.38% release at T1, confirming MGA’s effectiveness of MGA in delivering bioactive compounds to the intestinal mucosa. The controlled release provides a specific delivery of asiaticoside and asiatic acid to the intestinal mucosa, where they have anti-inflammatory functions: they inhibit NF-kB and upregulate the activities of antioxidants, namely, SOD and catalase [32]. This can be transposed physiologically into better enteric stress restriction, an issue in intensive broiler systems, and improved gut barrier integrity, which can reduce the incidence of coccidiosis by 10%–15% and consequently increase feed conversion ratios (FCR) by 58% [33]. Moderate water activity (a_w_ 0.604–0.670) and negative correlation with gizzard digestibility (r = −0.810) further validate this, as they minimize premature release in the acidic gizzard (pH 3), enhancing bioavailability, which is consistent with the 82.38% intestinal release data [33].

The composition of MGA enhances its appeal in poultry feed applications. MD provides a readily digestible carbohydrate source, contributing to energy availability, whereas GA’s natural surface activity and amphiphilic properties improve emulsion stability, ensuring a uniform distribution of phytoconstituents in feed pellets. The biocompatibility and widespread availability of these materials reduce production costs by 10%–15% compared to protein-based alternatives, such as gelatin, which require complex extraction processes [34,35]. For broilers, this sustained release of antioxidants may reduce lipid peroxidation in muscle tissue by 15%–20%, enhancing meat shelf life and color stability, which are key factors in meeting consumer demand for antibiotic-free products.

In contrast, protein-based materials such as albumen and gelatin offer superior TPC and antioxidant activity because of their dense, less porous matrices formed via hydrogen bonding and Maillard crosslinking during drying [36]. Their smooth, spherical morphology, as observed via SEM, reflects optimal film solidification, and their vulnerability to pepsin and trypsin results in higher release rates (75.3% and 78.6%, respectively) through proteolytic cleavage [37]. However, their high cost and processing complexity limit scalability. S. Alginate, despite its high glass transition temperature (T_m_ 104.55°C), showed poor EE (13.73%) and release (52.7%), likely due to its rigid, ionically crosslinked network, which resists enzymatic breakdown. PEG exhibited moderate stability (T_m_ 83.08± 8.38°C) but irregular morphology, indicating limited cohesive strength.

Thermal analysis via DSC confirmed MGA’s balanced stability of MGA (T_m_ 80.96±14.67°C), which is suitable for feed processing temperatures (70°C–90°C) without degrading bioactivity. This T_m_ value aligns with the observed thermal stability, supporting MGA’s practicality in industrial feed production. The intermediate roughness observed in SEM likely increases the surface area for enzymatic action, enhancing intestinal delivery compared with denser protein matrices [38]. FTIR spectral shifts (1,000–1,100 cm^−1^) suggest physical entrapment via hydrogen bonding, in contrast to covalent interactions in protein systems, and support a release mechanism driven by matrix swelling and erosion. This mechanism is consistent with 82.38% intestinal release, reinforcing MGA’s controlled-release advantage [38].

The increase in transition temperature (Tm) observed in some blend formulations compared with the pristine material suggests stronger intermolecular interactions and the formation of a more stable matrix structure. This may be due to hydrogen bonding, electrostatic attraction, or polymer–polymer compatibility among the encapsulating agents and the core material, which can promote tighter molecular packing and require more heat for thermal transition. In particular, materials such as S. Alginate, gelatin, and MD+GA may form denser networks that enhance structural order and thermal resistance. The higher Tm values, therefore, indicate that these blends produced microcapsules with improved thermal stability, which is desirable for processing and controlled-release applications.

The economic and practical implications of this study are thus significant. The high EE of MGA ensures consistent phytogenic delivery, reducing feed waste, whereas its intestinal release supports gut health and productivity, potentially reducing veterinary costs by 5%–8% annually [39]. These benefits are directly supported by the T1 (1:10) results, where high EE and digestibility indicated practical efficacy in broiler nutrition. Future *in vivo* trials should explore the impact of MGA on broiler gut microbiota (enrichment of *Lactobacillus* spp.) and meat oxidative stability, possibly by optimizing freeze-drying parameters to enhance thermal and release profiles. Integrating MGA with protein-based systems can leverage synergistic effects and balance cost and efficacy. In conclusion, MGA’s encapsulation performance of MGA, driven by its polysaccharide synergy, offers a sustainable and efficient solution for delivering *C. asiatica* phytoconstituents, enhancing broiler health, and supporting antibiotic-free production.

## CONCLUSION

The present study investigated the ethanolic extract of *C. asiatica* using different coating materials, such as MD+GA, PEG, egg albumen, gelatin, and S. Alginate, which can be utilized as PFAs for poultry nutrition. Wall materials composed of MD+ GA performed better than the other wall materials, and MD+ GA had excellent EE, TPC, antioxidant activities, and gastrointestinal release. The thermal stability of these formulations was adequate for feed processing, and their morphology exhibited a porous, sheet-like, and irregular structure, indicating successful embedding of the core material within the matrix, while DSC analysis suggested strong molecular interactions contributing to improved thermal stability. In contrast, S. Alginate was not very effective, despite its thermal stability. The higher stability of MD+GA demonstrates its potential to improve the stability, bioavailability, and controlled release of bioactive compounds from *C. asiatica* at specific targets. These results suggest that MD+GA encapsulation systems have potential as temperature-stable PFAs for supporting sustainable antibiotic-free poultry production and the subsequent supply of healthier and safer animal-derived food products globally (Figure 6).

## Figures and Tables

**Figure 1:**
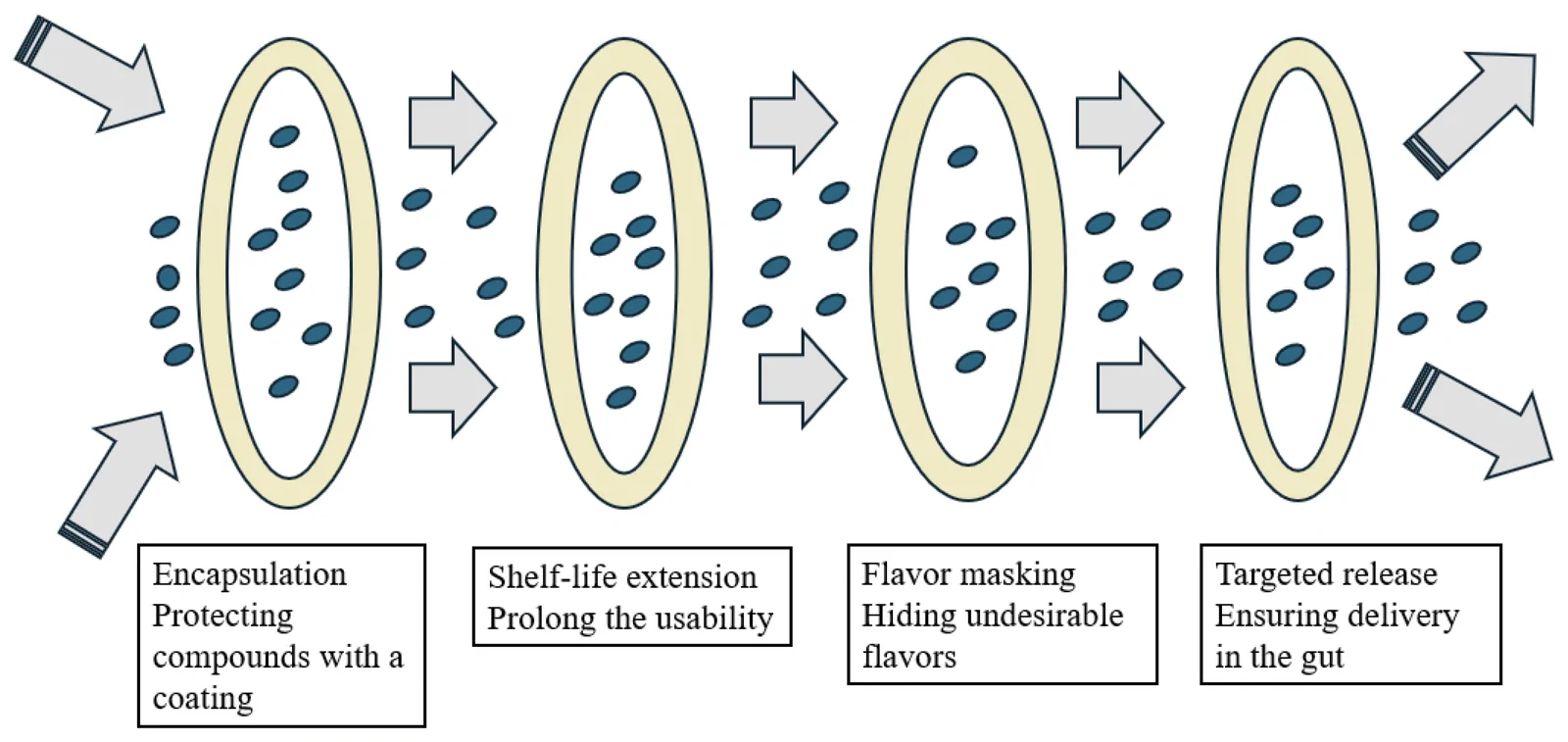
Schematic representation of microencapsulation highlighting protection of bioactive compounds, shelf-life extension, flavor masking, and targeted release in the gastrointestinal tract.

**Figure 2:**
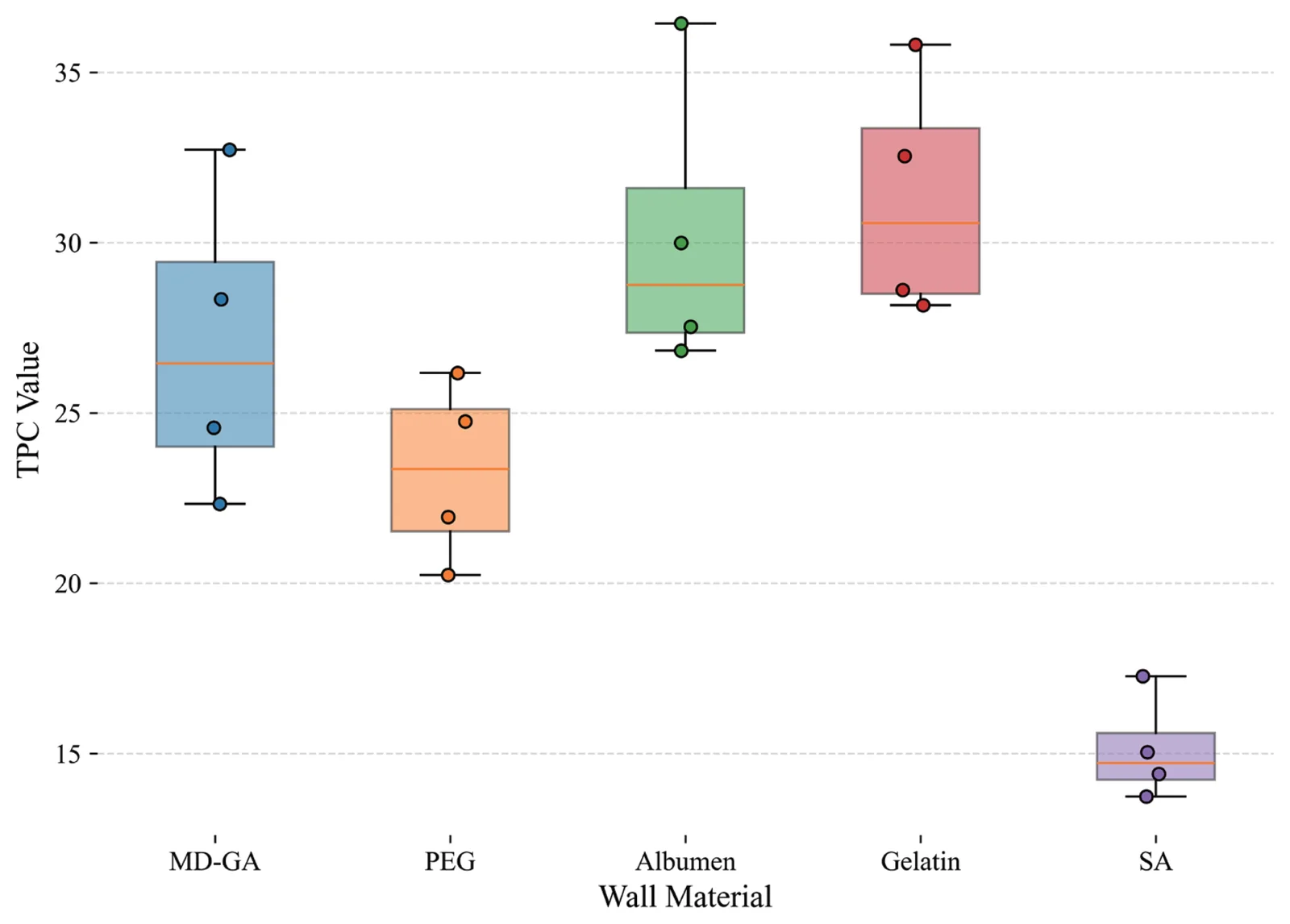
Total phenolic content (TPC; mg GAE/g) of microencapsulated *Centella asiatica* extract prepared with different wall materials. Values are presented as box plots with individual observations. T1–T4 represent core-to-wall ratios of 1:10, 1:8, 1:6, and 1:4, respectively. T5 was excluded from this analysis because it contained wall material only without *Centella asiatica* extract. MD_GA, maltodextrin+gum arabic; PEG, polyethylene glycol; SA, sodium alginate.

**Figure 3:**
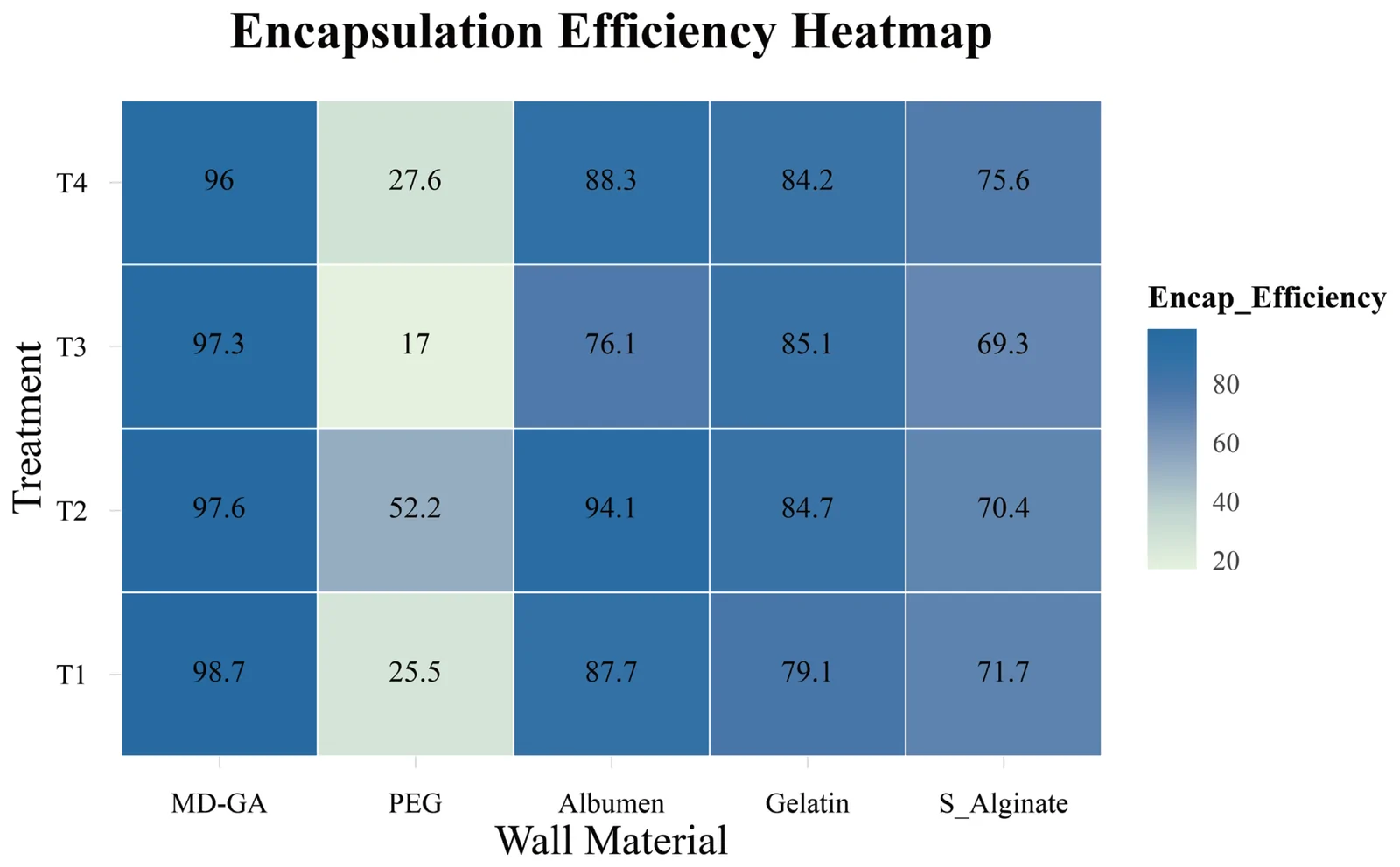
Encapsulation efficiency (%) of microencapsulated *Centella asiatica* extract prepared with different wall materials. T1–T4 represent core-to-wall ratios of 1:10, 1:8, 1:6, and 1:4, respectively. T5 was excluded from this analysis because it contained wall material only without *Centella asiatica* extract. MD_GA, maltodextrin+gum arabic; PEG, polyethylene glycol; S_Alginate, sodium alginate.

**Figure 4:**
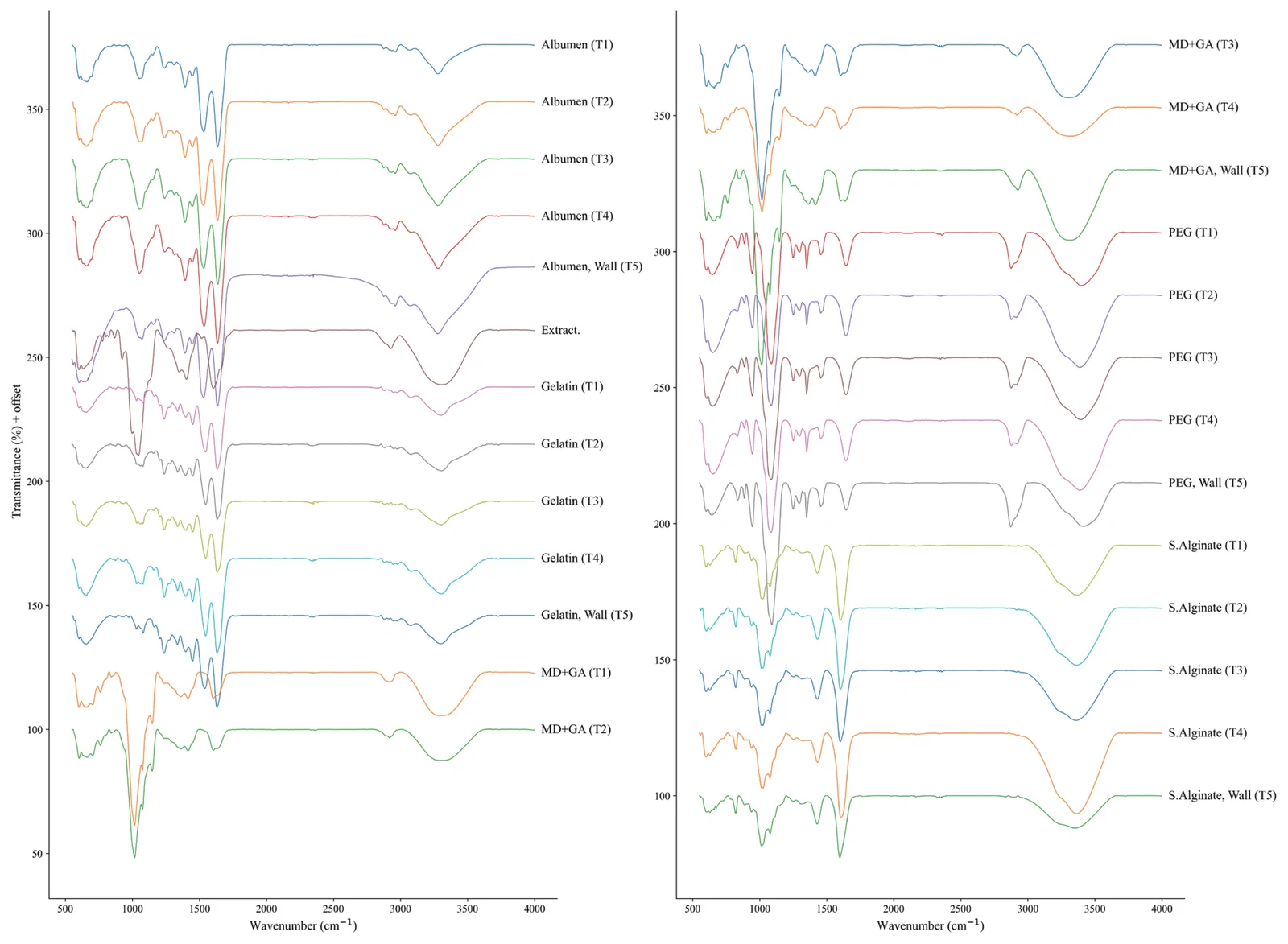
FTIR spectra of *Centella asiatica* encapsulation formulations using various wall materials. Core-to-wall material ratios; T1 = 1:10, T2 = 1:8, T3 = 1:6, T4 = 1:4, T5 = Only wall. MD+GA, maltodextrin+gum arabic; PEG, polyethylene glycol; S. Alginate, sodium alginate; FTIR, Fourier-transform infrared spectroscopy.

**Figure 5:**
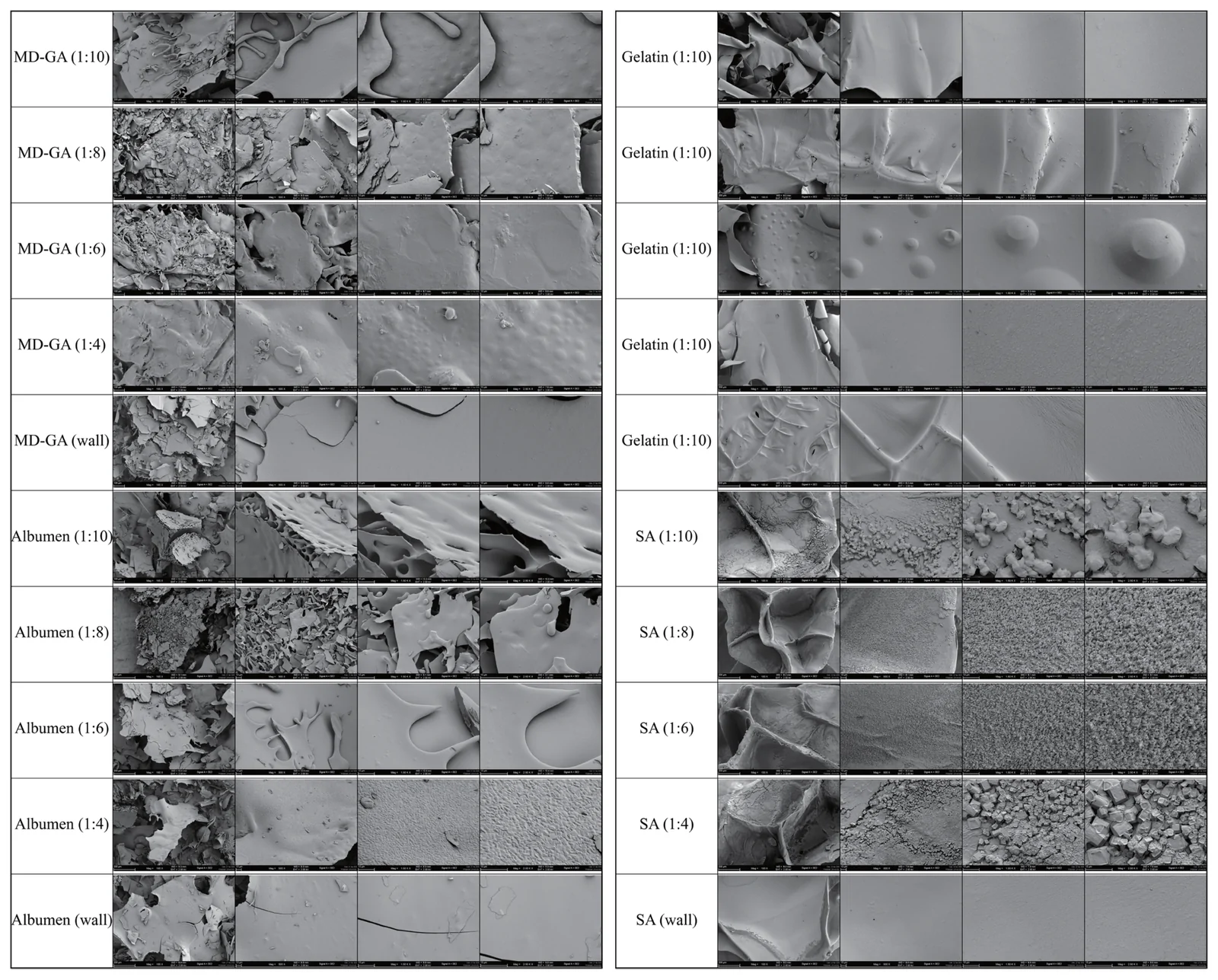
The scanning electron microscopy (SEM) images of *Centella asiatica* microcapsules with maltodextrin+gum arabic (MGA) as the wall material at core-to-wall ratios of (1:10, 1:8, 1:6, and 1:4) and using albumen as the wall material at core-to-wall ratios of (1:10, 1:6, and 1:4) and the wall-only (wall) condition. Each row displays several images of a particular ratio or wall-only sample to display the surface morphology and structural integrity, with MGA presenting rough, fragmented surfaces and albumen having smoother, looser structures depending on the ratio.

**Figure 6:**
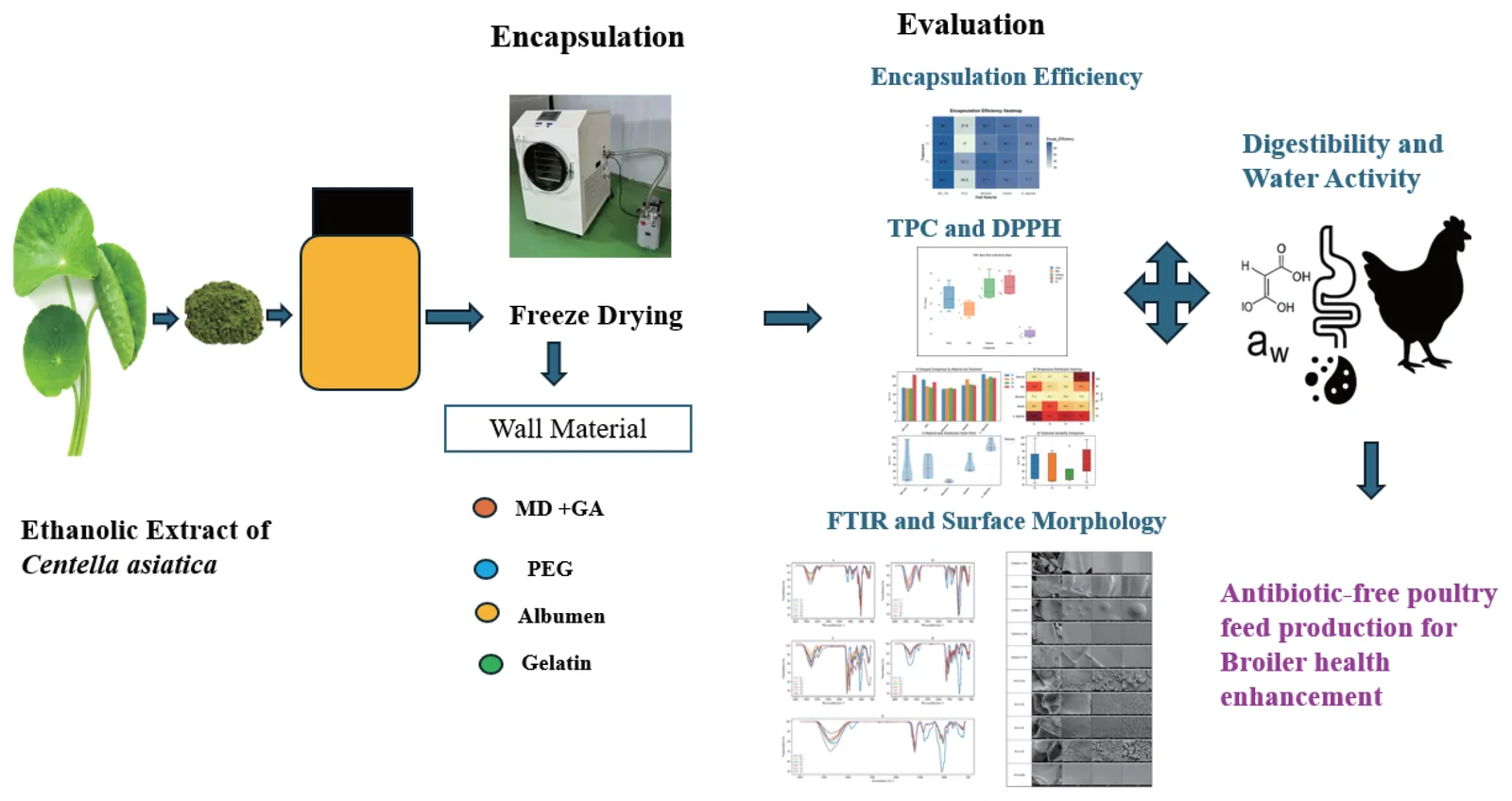
Microencapsulation of *Centella asiatica* ethanolic extract using various wall materials, its physicochemical characterization, and *in vitro* evaluation for antibiotic-free broiler feed application. MD+GA, maltodextrin+gum arabic; PEG, polyethylene glycol; TPC, total phenolic content; FTIR, Fourier-transform infrared spectroscopy.

**Table 1:** Illustrates the DPPH radical-scavenging activity (% inhibition) of microencapsulation wall materials for phytogenic feed additives in poultry

Treatment	MD+GA	PEG	Albumen	Gelatin	S. Alginate	Extract
T1	15.60±0.23^d^	17.03±0.26^c^	17.47±0.26^c^	19.58±0.29^c^	4.66±0.07^d^	8.60±0.13^d^
T2	16.30±0.24^c^	15.97±0.24^d^	16.20±0.24^d^	18.25±0.27^d^	6.88±0.10^c^	10.75±0.16^c^
T3	18.60±0.28^b^	19.47±0.29^b^	20.58±0.31^b^	25.79±0.39^b^	8.15±0.12^b^	14.28±0.21^b^
T4	20.50±0.31^a^	21.74±0.33^a^	22.07±0.33^a^	27.51±0.41^a^	9.10±0.14^a^	21.50±0.32^a^

Core-to-wall material ratios; T1 = 1:10, T2 = 1:8, T3 = 1:6, T4 = 1:4, T5 = Only wall. T5 was excluded from this analysis because it contained wall material only without *Centella asiatica* extract.

Columns represent different encapsulating materials or extract types and rows represent different treatments or formulations.

The ANOVA p-values for each condition were as follows: MD+GA (p ≈ 0.0001), PEG (p ≈ 0.0001), albumin (p ≈ 0.0001), gelatin (p ≈ 0.0001), S. Alginate (p ≈ 0.0001), and extract (p ≈ 0.0001), indicating significant differences among treatments for all conditions.

a–d Means with different superscripts in the same column indicate significant differences (p<0.05, Tukey’s HSD test).

MD+GA, maltodextrin+gum arabic; PEG, polyethylene glycol; S. Alginate, sodium alginate; ANOVA, analysis of variance; HSD, honest significant difference.

**Table 2:** The digestibility in the simulated intestine and thermal transition temperatures (T_m_, °C) of microencapsulation wall materials for phytogenic feed additives in poultry

Parameter	Treatment	MD+GA	PEG	Albumen	Gelatin	S. Alginate
Digestibility (%)	T1	82.38±1.24^a^	71.91±1.08^a^	82.75±1.24^b^	80.56±1.21^b^	74.22±1.11^b^
Digestibility (%)	T2	76.88±1.15^b^	72.40±1.09^a^	76.92±1.15^c^	75.46±1.13^c^	79.94±1.20^a^
Digestibility (%)	T3	72.46±1.09^c^	65.95±0.99^b^	84.59±1.27^a^	93.21±1.40^a^	67.48±1.01^d^
Digestibility (%)	T4	64.97±0.97^d^	63.85±0.96^c^	71.14±1.07^d^	72.82±1.09^d^	72.66±1.09^c^
(T_m_, °C)	T1	74.48±1.12^b^	92.76±1.39^a^	71.48±1.07^c^	80.17±1.20^c^	104.55±1.57^a^
(T_m_, °C)	T2	72.72±1.09^b^	77.65±1.16^c^	72.66±1.09^b^	93.22±1.40^a^	95.23±1.43^c^
(T_m_, °C)	T3	73.21±1.10^b^	74.79±1.12^d^	73.78±1.11^a^	81.62±1.22^b^	98.86±1.48^b^
(T_m_, °C)	T4	103.44±1.55^a^	87.10±1.31^b^	71.99±1.08^c^	79.99±1.20^c^	96.05±1.44^c^

Core-to-wall material ratios; T1 = 1:10, T2 = 1:8, T3 = 1:6, T4 = 1:4, T5 = Only wall. T5 was excluded from this analysis because it contained wall material only without *Centella asiatica* extract.

Values represent the mean±standard deviation (SD) of three replicates.

a–d Superscripts indicate significant differences between treatments within each column, determined by one-way ANOVA followed by Tukey’s HSD post-hoc test (p<0.05). Means with different superscripts in the same column are significantly different.

ANOVA p-values for each condition were as follows: MD+GA (p ≈ 0.0001), PEG (p ≈ 0.0001), albumin (p ≈ 0.0001), gelatin (p ≈ 0.0001), and S. Alginate (p ≈ 0.0001), indicating significant differences among treatments for all conditions.

MD+GA, maltodextrin+gum arabic; PEG, polyethylene glycol; S. Alginate, sodium alginate; ANOVA, analysis of variance; HSD, honest significant difference.

**Table 3:** The water activity (a_w_) of *Centella asiatica* microencapsulation wall materials (maltodextrin with gum arabic, polyethylene glycol, albumen, gelatin, sodium alginate) for phytogenic feed additives

Treatment	MD+GA	PEG	Albumen	Gelatin	S. Alginate
T1	0.66±0.01^b^	0.86±0.01^d^	0.36±0.01^c^	0.39±0.01^b^	0.16±0.00^a^
T2	0.66±0.01^c^	0.88±0.01^c^	0.34±0.01^d^	0.42±0.01^a^	0.12±0.00^c^
T3	0.67±0.01^a^	0.88±0.01^a^	0.37±0.01^b^	0.39±0.01^b^	0.13±0.00^b^
T4	0.62±0.01^d^	0.88±0.01^b^	0.44±0.01^a^	0.42±0.01^a^	0.10±0.00^d^

Core-to-wall material ratios; T1 = 1:10, T2 = 1:8, T3 = 1:6, T4 = 1:4, T5 = Only wall. T5 was excluded from this analysis because it contained wall material only without *Centella asiatica* extract.

Values represent the mean±standard deviation (SD) of three replicates.

a–d Means with different superscripts in the same column indicate significant differences (p<0.05, Tukey’s HSD test).

MD+GA, maltodextrin+gum arabic; PEG, polyethylene glycol; S. Alginate, sodium alginate; HSD, honest significant difference.

**Table 4:** Color parameters (L^*^, a^*^, b^*^) of *Centella asiatica* microcapsules prepared using different wall materials (maltodextrin with gum arabic, polyethylene glycol, albumen, gelatin, and sodium alginate) for phytogenic feed additives

Treatments	MD+GA			PEG			Albumen			Gelatin			S. Alginate		
				
	L^*^	a^*^	b^*^	L^*^	a^*^	b^*^	L^*^	a^*^	b^*^	L^*^	a^*^	b^*^	L^*^	a^*^	b^*^
T1	79.43 ±0.60^b^	−0.50 ±0.10^c^	15.58 ±0.34^d^	78.43 ±1.40^b^	−0.47 ±0.12^c^	15.24 ±0.24^d^	79.84 ±0.29^a^	−2.17 ±0.06^b^	15.46 ±0.32^d^	69.57 ±0.49^a^	−1.34 ±0.03^b^	8.16 ±0.02^c^	46.53 ±0.35^b^	0.59 ±0.02^a^	16.40 ±0.26^c^
T2	76.49 ±0.38^c^	−0.46 ±0.01^c^	17.54 ±0.02^c^	75.15 ±0.88^c^	−0.49 ±0.05^c^	16.56 ±1.75^c^	79.18 ±0.33^a^	−1.95 ±0.05^c^	17.47 ±0.23^c^	65.17 ±0.06^b^	−1.12 ±0.04^b^	5.83 ±0.07^d^	45.33 ±0.20^b^	0.36 ±0.01^b^	15.50 ±0.40^c^
T3	61.57 ±0.21^d^	0.98 ±0.01^b^	20.30 ±0.26^b^	62.23 ±0.38^d^	0.97 ±0.02^b^	20.50 ±0.72^b^	79.19 ±0.23^a^	−2.25 ±0.03^a^	17.55 ±0.02^c^	63.32 ±0.29^c^	−1.56 ±0.02^a^	9.47 ±0.08^b^	45.42 ±0.19^b^	0.32 ±0.01^b^	17.34 ±0.20^b^
T4	59.88 ±0.69^d^	1.37 ±0.01^a^	24.41 ±0.21^a^	60.35 ±1.18^d^	1.36 ±0.02^a^	23.95 ±0.37^a^	79.52 ±0.43^a^	−1.27 ±0.07^d^	21.18 ±0.08^a^	58.59 ±0.42^d^	−1.30 ±0.01^b^	10.39 ±0.23^a^	49.41 ±0.27^b^	−0.07 ±0.01^c^	19.19 ±0.27^a^
T5	85.90 ±0.76^a^	−0.11 ±0.01^c^	2.53 ±0.38^e^	84.90 ±0.24^a^	−0.10 ±0.02^c^	2.13 ±0.06^e^	86.58 ±0.45^b^	−1.17 ±0.02^d^	10.34 ±0.01^b^	69.47 ±0.32^a^	−0.22 ±0.01^c^	1.30 ±0.23^e^	70.37 ±0.25^a^	−0.49 ±0.02^d^	11.26 ±0.02^d^

Core-to-wall material ratios: T1 = 1:10, T2 = 1:8, T3 = 1:6, T4 = 1:4, T5 = only wall.

Values represent the mean±standard deviation (SD) of three replicates.

a–d Different superscripts within the same column indicate significant differences (p<0.05, Tukey’s HSD test).

MD+GA, maltodextrin+gum arabic; PEG, polyethylene glycol; S. Alginate, sodium alginate; HSD, honest significant difference.

## Data Availability

Upon reasonable request, the datasets of this study can be available from the corresponding author.

## SUPPLEMENTARY MATERIAL

**Supplement 1**. Core-to-wall material ratios in various treatments at each of the five encapsulation tests.

**Supplement 2**. The encapsulation efficiency (%) of microencapsulation wall materials for phytogenic feed additives in poultry.

**Supplement 3**. Correlation matrix of *Centella asiatica* microcapsules across treatments showing relationships between Total Phenolic Content (TPC), Encapsulation Efficiency (EE), DPPH inhibition, Transition Temperature (T_m_), Gizzard Digestibility, Intestinal Digestibility, and Water Activity (a_w_).
