# Supplementary material for: Extending the use of Centella asiatica extract in poultry applications using distinct freeze-drying wall materials
Source: Anim Biosci. 2026 Jul 27;39(10):260300. doi: 10.5713/ab.260300 (PMC13662943; doi:10.5713/ab.260300)
Supplement: Supplementary file 1 [file ab-260300-Supplementary-1.pdf]

Supplement 1. Core-to-wall material ratios in various treatments at each of the five encapsulation tests

Treatment	MD+GA	PEG	Albumen	Gelatin	S. Alginate
T1	1:10	1:10	1:10	1:10	1:10
T2	1:8	1:8	1:8	1:8	1:8
T3	1:6	1:6	1:6	1:6	1:6
T4	1:4	1:4	1:4	1:4	1:4
T5	Only wall	Only wall	Only wall	Only wall	Only wall

MD+GA (Maltodextrin + Gum Arabic), PEG (Polyethylene Glycol), Albumen, Gelatin, and S. Alginate (Sodium Alginate).
