# Supplementary material for: Extending the use of Centella asiatica extract in poultry applications using distinct freeze-drying wall materials
Source: Anim Biosci. 2026 Jul 27;39(10):260300. doi: 10.5713/ab.260300 (PMC13662943; doi:10.5713/ab.260300)
Supplement: Supplementary file 2 [file ab-260300-Supplementary-2.pdf]

Supplement 2. The encapsulation efficiency (%) of microencapsulation wall materials for phytogenic feed additives in poultry

Treatment	MD+GA	PEG	Albumen	Gelatin	S. Alginate
T1	98.67 ± 0.47^{a}	25.47 ± 0.25^{c}	87.69 ± 0.88^{b}	79.13 ± 0.79^{b}	71.71 ± 0.72^{b}
T2	97.62 ± 0.48^{b}	52.16 ± 0.52^{a}	94.14 ± 0.94^{a}	84.69 ± 0.85a	70.38 ± 0.70^{c}
T3	97.27 ± 0.48^{b}	16.98 ± 0.17^{d}	76.08 ± 0.76^{c}	85.12 ± 0.85^{a}	69.27 ± 0.69^{d}
T4	96.00 ± 0.48^{c}	27.59 ± 0.28^{b}	88.28 ± 0.88^{b}	84.16 ± 0.84^{a}	75.56 ± 0.76^{a}

MD+GA (Maltodextrin + Gum Arabic), PEG (Polyethylene Glycol), Albumen, Gelatin, and S. Alginate (Sodium Alginate).

Core-to-wall material ratios; T1 = 1:10, T2 = 1:8, T3 = 1:6, T4 = 1:4, T5 =Only wall. T5 was excluded from this analysis because it contained wall material only without *Centella asiatica* extract.Means with different superscripts in the same column indicate significant differences ($p < 0.05$, Tukey's HSD test). ANOVA p-values: MD+GA ($p \approx 0.0001$), PEG ($p < 0.0001$), albumin ($p \approx 0.0001$), gelatin ($p \approx 0.0001$), and S. Alginate ($p \approx 0.0001$).
