# Supplementary material for: Extending the use of Centella asiatica extract in poultry applications using distinct freeze-drying wall materials
Source: Anim Biosci. 2026 Jul 27;39(10):260300. doi: 10.5713/ab.260300 (PMC13662943; doi:10.5713/ab.260300)
Supplement: Supplementary file 3 [file ab-260300-Supplementary-3.pdf]

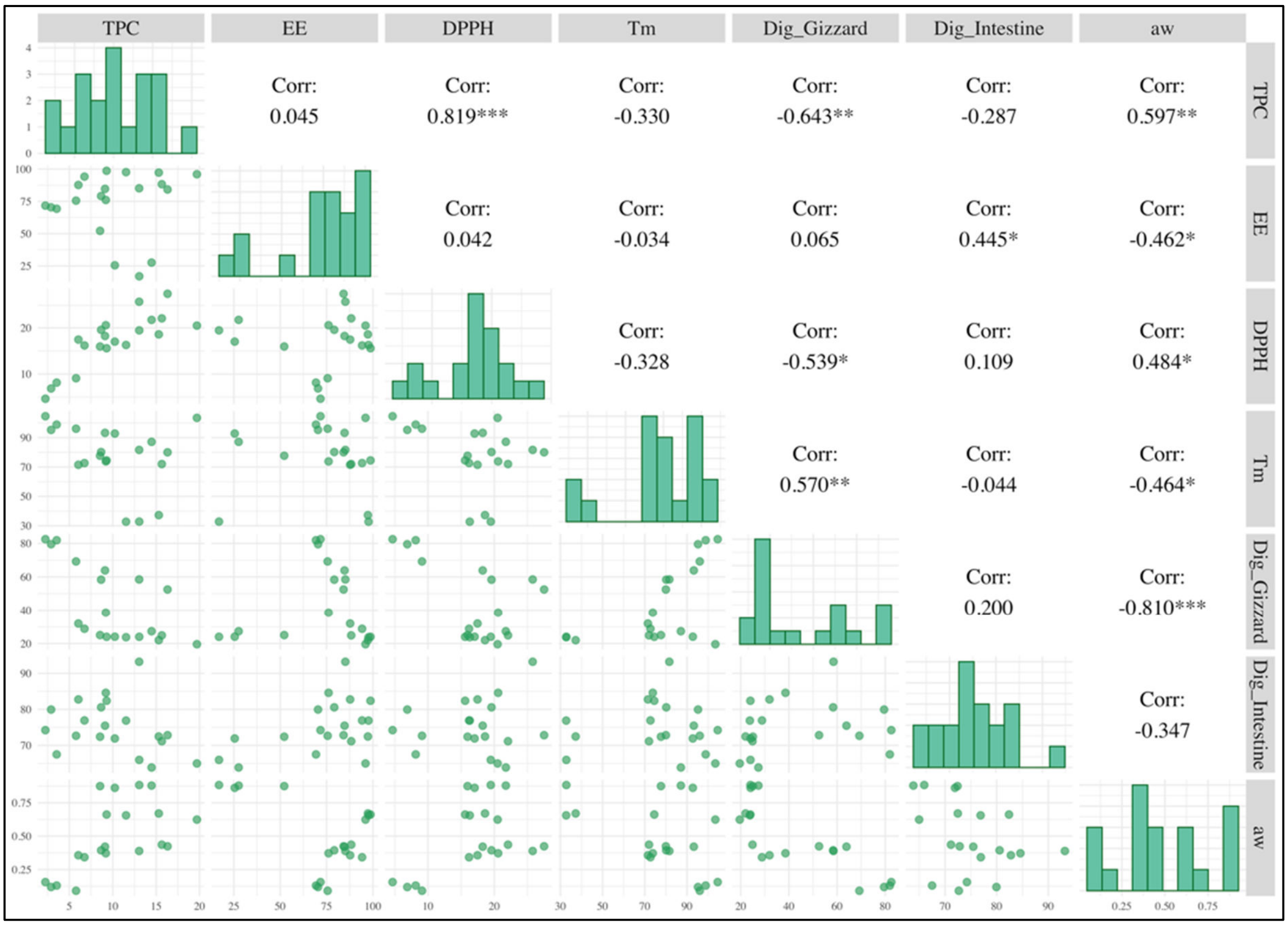

Supplement 3. Correlation matrix of *Centella asiatica* microcapsules across treatments showing relationships between Total Phenolic Content (TPC), Encapsulation Efficiency (EE), DPPH inhibition, Transition Temperature (T_m), Gizzard Digestibility, Intestinal Digestibility, and Water Activity (a_w). Significant correlation coefficients are marked (* $p < 0.05$, ** $p < 0.01$, *** $p < 0.001$). A strong positive correlation (TPC and EE, r = 0.819) is depicted in green, while a negative correlation (T_m and a_w, r = −0.348) is shown, with scatter plots illustrating the data distributions.
